# *intSDM*: An *R* Package for Building a Reproducible Workflow for the Field of Integrated Species Distribution Models

**DOI:** 10.1002/ece3.71029

**Published:** 2025-03-13

**Authors:** Philip S. Mostert, Ragnhild Bjørkås, Angeline J. H. M. Bruls, Wouter Koch, Ellen C. Martin, Sam W. Perrin

**Affiliations:** ^1^ Department of Mathematical Sciences Norwegian University of Science and Technology Trondheim Norway; ^2^ Centre for Biodiversity Dynamics Norwegian University of Science and Technology Trondheim Norway; ^3^ Norwegian Biodiversity Information Centre Trondheim Norway; ^4^ Gjærevollsenteret Norwegian University of Science and Technology Trondheim Norway

**Keywords:** citizen science, data integration, integrated species distribution model, reproducible workflow, spatial point process

## Abstract

There has been an exponential increase in the quantity and type of biodiversity data in recent years, including presence‐absence, counts, and presence‐only citizen science data. Species Distribution Models (SDMs) have typically been used in ecology to estimate current and future ranges of species and are a common tool used when making conservation prioritization decisions. However, the integration of these data in a model‐based framework is needed to address many of the current large‐scale threats to biodiversity. Current SDM practice typically underutilizes the large amount of publicly available biodiversity data and does not follow a set of standard best practices. Integrating different data types with open‐source tools and reproducible workflows saves time, increases collaboration opportunities, and increases the power of data inference in SDMs. We aim to address this issue by (1) proposing methods and (2) generating a reproducible workflow to integrate different available data types to increase the power of SDMs. We provide the *R* package *intSDM*, as well as guidance on how to accommodate users' diverse needs and ecological questions with different data types available on the Global Biodiversity Information Facility (GBIF), the largest biodiversity data aggregator in the world. Finally, we provide a case study of the application of our proposed reproducible workflow by creating SDMs for vascular plants in Norway, integrating presence‐only and presence‐absence species occurrence data as well as climate data.

## Graphical abstract

There has been an exponential increase in heterogeneous biodiversity data, each with its own sampling protocols, and advantages and disadvantages. We provide an R package designed to assist users in constructing a workflow to use these data effectively in a single statistical framework.

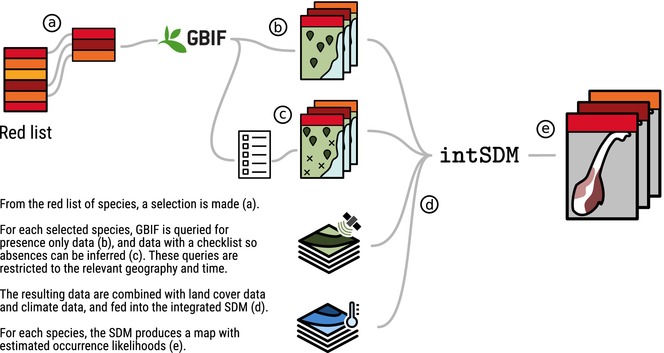

## 1 Introduction

There has been an unprecedented increase in biodiversity information over the last decade as a result of the exponential rise in digital technology to collect and store data effectively (Michener and Jones 2012). Analyzing this massive collection of data has the potential to help us in our efforts to answer a multitude of ecological questions on a far larger scale than could previously be achieved.

A major difficulty in employing these data at large, however, lies in the heterogeneity of the individual datasets collected, making combining them into a single modeling framework, notably species distribution models (SDMs), challenging (Kelling et al. 2009; König et al. 2019; Simmonds et al. 2020). Individual datasets are often collected with a specific purpose in mind, leading to different spatio‐temporal resolutions, sampling protocols, and variable names between each dataset. A substantial amount of available data is collected by citizen scientists, which is often opportunistic in nature and known to contain a multitude of different biases (Sicacha‐Parada et al. 2021). As a result, significant care needs to be taken when combining these datasets; neglecting the biases when analyzing them could lead to poor inference (Simmonds et al. 2020).

Integrated species distribution models (ISDMs) have been proposed as a solution to the problem of data heterogeneity: allowing researchers to combine data from disparate sources under a single modeling framework by assuming that each is derived from a common underlying distribution (Heberling et al. 2021; Isaac et al. 2020). A multitude of benefits has been found from implementing these models. Initially, they were used to show that the effect of biases typical in opportunistic occurrence records may be reduced by both including covariates and flexible spatial terms to account for the sampling biases when analyzing them in conjunction with structured survey data (Dorazio 2014; Simmonds et al. 2020). Furthermore, these models expand the scope or scale of interest, increase the precision and accuracy of models, improve inference about potential distribution shifts of organisms in space and time, and allow data use to extend beyond original intent at the time of collection, blending different sampling methods and dataset properties (Dorazio 2014; Fithian et al. 2015; Miller et al. 2019; Simmonds et al. 2020).

Typical ISDM analyses are tailored towards small‐scale studies and only use a subset of all data available. Despite this, the tools and computational resources needed to analyze big data using data‐integration methods on a large scale are certainly accessible and available today (Farley et al. 2018). Additionally, no current implementation of these models does so in a coherent workflow, documenting the relevant parts of the process. We therefore provide the tools and recommendations for developing and documenting a workflow required to estimate large‐scale ISDMs in an automated and reproducible framework. The implementation of our proposed workflow is available through the *R* package (R Core Team 2023) *intSDM*, which is designed to combine structured and unstructured data available on GBIF (Global Biodiversity Information Facility, the largest biodiversity data aggregator in the world).

Procedures and steps required to build reproducible workflows and pipelines to transform raw data to obtain periodic estimates of species occupancy have recently been described by both Boyd et al. (2023) and Cervantes et al. (2023). We follow the structure of their workflows in a similar fashion, but put emphasis on the methods regarding using multiple disparate datasets and estimating large‐scale integrated SDMs.

Herein we report a case study of the application of our reproducible workflow to create SDMs of vascular plants in Norway by integrating two sources of information: presence‐only and presence‐absence data using the *intSDM R* package. A detailed analysis of the case study is also provided as a vignette within the *R* package.

## 2 Methods

### 2.1 *intSDM* R Package

Our proposed workflow is presented through the *R* package, *intSDM*. The package is designed to construct ISDMs at a large scale by following the conceptual workflow steps described as follows:

1. Obtain heterogeneous occurrence data and covariate layers.
2. Process, clean, and filter data.
3. Fit an integrated model for the data.
4. Preform model assessment and selection.
5. Create summaries and communicate results.

**FIGURE 1:**
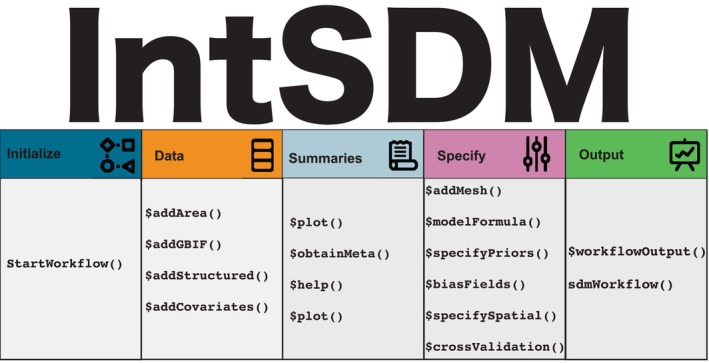
An overview of the functions used in the R package intSDM. These functions are categorized by one of five steps related to the construction of the workflow: Initialization, data, summaries, options, or output.

This subsection is dedicated to describing the functions of the package, as well as the considerations practitioners should take when developing these types of workflows. An overview of the toolkit available within the *intSDM* R package is provided in Figure 1.

The package is currently available on both CRAN and github, and may therefore be downloaded using either one of the following scripts:install.packages(‘intSDM’).*#or*devtools::install_github(‘PhilipMostert/intSDM’).


### 2.2 Functionality of Package

The first function considered in the package is startWorkflow, which initializes the workflow. The arguments of this function are designed to specify non‐model components of the workflow, such as the names of the species considered in the workflow and the study domain, as well as other project options such as directories and project names.

The default for this function is to create estimates for each species independently; however, setting the argument Richness = TRUE estimates a multi‐species model. This allows the user to obtain maps of estimated species richness. An example of an analysis estimating species richness using the *intSDM R* package is presented as a vignette within the package.

The output from this function is an *R6* (Chang 2021) object containing different slot functions (which are represented by the ‘.$functionName’ notation) used to customize the different components of the workflow, breaking it down into manageable chunks. These slot functions are illustrated in Figure 1, and are related to different parts of the workflow. Documentation and help related to these slot functions is easily obtained by calling. $help.

Once all the parts of the workflow have been specified, the outputs of the workflow may be obtained using the function sdmWorkflow. This function will return either a list of model outputs or save it to a pre‐defined directory, depending on the Save argument in startWorkflow.

#### 2.2.1 Identify and Process Data

Data are fundamental for our workflow, and two types of data are necessary to estimate species' distributions: geolocated observation data detailing the sampling locations for the species across some geographic domain, and environmental covariate layers describing the environmental conditions at each location across the same domain.

Observation data may be obtained from numerous different online repositories, such as *eBird* (Sullivan et al. 2014) or *iNaturalist* (Nugent 2018). Within our setting, we focus on GBIF (GBIF 2023) as it serves as an aggregated entry point for these and many other data sources.

This repository hosts a massive amount of data derived from a multitude of different data collectors and institutions using a variety of different sampling protocols in their collection process—although the data are mostly opportunistically collected citizen science records (Amano et al. 2016). Even though these observations are known to contain biases, their use in research is increasing, and they remain a vital source of information to supply into SDMs (Feldman et al. 2021).

Data from GBIF may be added to our workflow using the .$addGBIF function. The function's arguments are related to specifying the observation process of the dataset (one of presence‐only, presence‐absence or counts), as well as additional arguments to filter and process the data from GBIF using tools from the *rgbif* (Chamberlain et al. 2022) package (see? rgbif::occ_data for the filtering options available).

A primary objective in this step is separating the different occurrence datasets into different observation processes based on the sampling protocol considered in the collection protocol. This information is often known and can be located within the datasets' metadata file (Isaac and Pocock 2015). The observation process for a given dataset is specified in the workflow using the function's *datasetType* argument.

However, the most important part of using GBIF‐obtained data in a large‐scale workflow is that it is FAIR (findable, accessible, interoperable, and reusable) (Wilkinson et al. 2016) and follows data standards set by Biodiversity Information Standards (International Working Group on Taxonomic Databases 2006), such as Darwin Core, Ecological Metadata Language, and the Biological Collection Access Service standards (Heberling et al. 2021), making it easy to obtain and standardize in an automated procedure.

Data sharing of raw data on large online repositories is a key ingredient for data integration and has become more prevalent across ecology over the last decade (Culina et al. 2020). Despite this, not all data are readily available for open research (König et al. 2019; Michener and Jones 2012). As a result, researchers may need to obtain data from secondary locations. Sometimes occurrence data may be obtained from other sources such as country‐ or institution specific databases or through collaboration efforts (Michener and Jones 2012). These data would typically be high‐quality survey data and thus a valuable asset in conjunction with the citizen science records if the researcher is able to obtain them.

Non‐*GBIF* data may be added to the workflow using $addStructured. The use of the function is similar to that of $addGBIF, with arguments related to specifying the observation process for the data, as well as specifying the names of the response and the explanatory variables within each dataset.

In addition to observation data, this step of the workflow includes compiling environmental, habitat, or other variable data to be included in the models. They are typically collected via remote sensing or geographic information system (GIS) methods; possible sources of such data include WorldClim (Fick and Hijmans 2017), Copernicus (Copernicus 2023), CHELSA (Karger et al. 2017), and Light Detection and Ranging (LiDAR). These variables need to be selected with care prior to any analysis, being considerate of both the goals of the model and the underlying biology of the species studied (Araújo and Peterson 2012).

WorldClim and ESA land cover (Zanaga et al. 2022) environmental layers may be added to the workflow using the $addCovariates function. The arguments here are used to specify the name of the covariate, resolution, and function to apply to the data. Like the species occurrence data, environmental data other than WorldClim may be preferable. These environmental layers may be added using the function's *Object* argument.

#### 2.2.2 Data Documentation

Obtained data needs to be documented in order for it to be reproducible. This becomes a critical step as data are combined for use in analysis, and terms of use and quality control vary. Adequate data documentation is a key component of methods reproducibility (Zurell et al. 2020), and metadata standards have been proposed to facilitate tracking information on data authorship, preparation (i.e., cleaning, changing, or validating), and model estimation (Merow et al. 2019). Given the wide range of species' observational data and environmental data available for inclusion in SDMs, properly documenting the sources, metadata, and scales are necessary for reproducibility. Documenting data discovery and inclusion is also necessary to assess the comparative value of SDMs when different datasets are used to model the distribution of the same species.

Obtaining metadata from the workflow may be done using the $obtainMeta function. This function provides the citations for the WorldClim covariates and each of the GBIF‐obtained datasets used in the analysis.

#### 2.2.3 Customization of the Model

The model structure of the ISDM may be customized with some of the slot functions. The functions $specifySpatial and $specifyPriors allow the user to specify the priors for the spatial effects and the fixed and random effects, respectively. Information regarding penalizing complexity (PC) priors for the spatial effect may be found in Fuglstad et al. (2019), and information regarding Bayesian statistics may be found in Gelman et al. (2013).

The function $modelFormula allows for two arguments, *modelFormula* and *biasFormula*, to assist in specifying the formula for the covariates associated with the process‐level model and the covariates associated with the presence‐only observation models used to account for sampling biases, respectively. If *modelFormula* is not specified, then the covariates are included as linear terms.

#### 2.2.4 Estimation of the Integrated Species Distribution Model

Applied SDM use is now well established, and there are a multitude of different methods to fit these models (see for example, Fletcher and Fortin (2018)). Given the novelty of the field of ISDMs, bespoke *R* packages and software to fit the models appropriately are limited. Implementation of these models in the past has typically been done by assuming a point process framework and by estimating a joint‐likelihood through either maximum likelihood estimation (Dorazio 2014; Fithian et al. 2015; Koshkina et al. 2017) or through Bayesian models using Markov chain Monte Carlo (MCMC) techniques (Miller et al. 2019; Zulian et al. 2021). However, there are a variety of different methods to fit ISDMs; for example: combining results from independent models, treating the results from one model as a covariate in another, using one dataset to derive an informed prior for another in a Bayesian setting, or by simply pooling the datasets together (Fletcher Jr. et al. 2019).

The *PointedSDMs R* package (Mostert and O'Hara 2023) has been proposed to estimate these complex models in an easy‐to‐use framework. *PointedSDMs* is built around the *R‐INLA* package (Martins et al. 2013), which uses the integrated nested Laplace approximation (INLA) methodology (Rue et al. 2009) to approximate Bayesian latent Gaussian models. INLA is preferred to traditional Bayesian approximation techniques for continuous space models for its speed and computational efficiency in estimating Gaussian random fields (in the form of a Gaussian Markov random field) via the stochastic partial differential equation (SPDE) approach (Lindgren et al. 2011), which are used to account for unmeasured covariates and potential autocorrelations. Briefly, the INLA‐SPDE approach approximates the continuous process through a discretely indexed process based on a triangulated mesh built around the area of interest. Further details on constructing a mesh may be found in (Krainski et al. 2018) and (Lindgren and Rue 2015). *intSDM* uses *inlabru* (Bachl et al. 2019), an *R* package that builds wrapper functions around *R‐INLA* to assist in the construction and estimation of spatial point process models.

Our integrated model is defined as in Isaac et al. (2020), where the hierarchical state‐space model is the combination of a process model, used to reflect the “true” distribution of the species, with separate observation models for each of the datasets, where the likelihood for each of these models is dependent on the underlying sampling protocol of the corresponding dataset. Here, we treat the species' “true” distribution as an inhomogeneous Poisson process, with an intensity function given by $\lambda \left(s\right)=e^{\eta \left(s\right)}$, which describes the expected number of species at some point s. The log of this intensity is given by:

(1) $$\eta \left(s\right)=\alpha +\sum_{u=1}^{k}\;\beta_{u}X_{u}\left(s\right)+\zeta \left(s\right),$$

where: α is an intercept term, $X_{u}\left(s\right)$ are spatially varying environmental variables with corresponding coefficients $\beta_{u}$, and $\zeta \left(s\right)$ is a spatially varying Gaussian random field used to account for unmeasured covariates and potential spatial autocorrelation in the model. This linear predictor may be extended by adding variables to reflect biases or additional random fields. As a result, the expected number of species across some area Ω is therefore given by:

(2) $$\begin{array}{l} \mu \left(s\right)=\int_{\Omega }\lambda \left(s\right)\mathrm{ds}. \end{array}$$

The integral defined in Equation (2) is intractable, and as a result, needs to be approximated numerically using integration points (Simpson et al. 2016). These points are generated at the nodes of the spatial mesh using tools available from the *fmesher R* (Lindgren 2024) package.

The observation processes for our state‐space model depend on the type of data at hand, which typically come in the form of count data, presence‐absence or presence‐only data. Following Isaac et al. (2020) we model our count data in this framework as a Poisson point process with expected value given by the integral of the intensity function over the study area, and the presence‐absence data with a Binomial likelihood with a *cloglog* link function, which allows us to relate the probability of occurrence of the species to the log of the intensity function. Given that citizen science data are often collected opportunistically and may contain a multitude of different sampling biases (Sicacha‐Parada et al. 2021), we model the presence‐only data from a thinned Poisson process to account for imperfect detection in the collection process. As a result, we assume that the observed point process has intensity $\lambda \left(s\right)q\left(s\right)$, where $q\left(s\right)$ is the thinning probability, which may be specified using observational covariates or a second spatial effect.

#### 2.2.5 Validation and Assessment of Models

Validation of statistical models using independent data is vital in assessing model performance. However, model assessment and selection for ISDMs is incredibly difficult to complete since methods such as cross‐validation and variable selection may not perform well under this framework (Zipkin et al. 2021).

Nevertheless, validation of results and model comparison is an important step in any scientific analysis. Therefore our workflow incorporates two types of cross‐validation: a spatial‐block cross‐validation using functionality from the *blockCV* R package (Valavi et al. 2019) as well as a leave‐one–one cross‐validation (see for example [Mostert and O'Hara 2023]). Cross‐validation for the workflow may be specified by using. $crossValidation.

#### 2.2.6 Model Summaries and Outputs

The outputs of the ISDMs give us an understanding of how the underlying environment drives species richness and distribution across an area and are typically applied to some form of biodiversity assessment (such as selecting areas for restoration and protection).

These results can then be used to make future projections on which species are most sensitive to what environmental conditions, and how the changes in these conditions could force range shifts across time. Such knowledge could be useful for example, for management practitioners when identifying important areas for the protection of biodiversity in the future, or when assessing the risks of biological invasions (Guisan et al. 2013).

A popular output from these types of models is prediction maps of the intensity function of the model, which gives a reflection of the occurrence rate or the intensity of the species across a map. A useful way to extend the outputs of these models is by adding a temporal component to the model, which could reflect how this intensity changes over a time period.

Outputs from our model may be chosen using the $workflowOutput function, which allows the user to specify different outputs from the workflow. These include obtaining the *R‐INLA* model, predictions of the intensity function of the model, maps of the intensity function, and cross‐validation results.

### 2.3 Motivating Case Study

**FIGURE 2:**
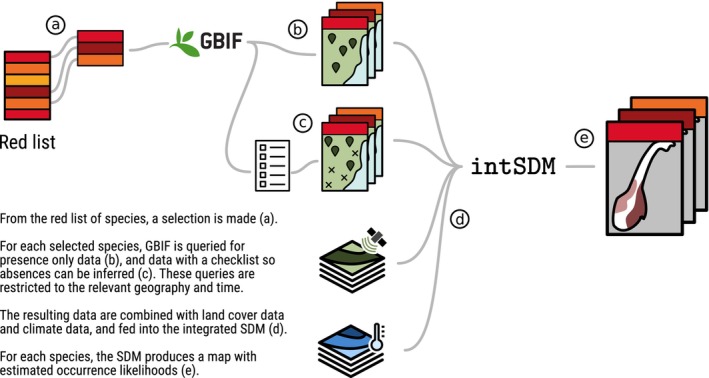
Visual summary of the workflow considered for our case study. The steps begin with a selection of species (a), a GBIF query of presence‐only data (b) and presence‐absence data (c), and a combination of covariate data (d). The package intSDM is used in the final step to estimate occurrence likelihoods (e).

As a case study, we used our reproducible workflow for integrating disparate datasets to create a species distribution map for vascular plant species in Norway (this particular workflow is illustrated in Figure 2). Such a map could be used for several purposes, for instance when locating new areas to protect for conservation. According to the Norwegian Red List of 2021, 22% of vascular plant species in Norway are considered threatened, that is, placed in the Red List categories Critically Endangered (CE), Endangered (EN) or Vulnerable (VU) (Norwegian Biodiversity Information Centre 2021). A major goal when designating new protected areas, both in Norway (Norwegian Biodiversity Information Centre 2021) and globally (International Union for Conservation of Nature 2022) is to protect species diversity and prevent extinction. It is essential to be able to accurately map the distribution of threatened species to locate areas of importance. To do this, all available data in various standards and qualities must be combined in an effective way.

For this example, we obtained presence‐only data published by the Norwegian Biodiversity Centre through GBIF (The Norwegian Biodiversity Information Centre 2023). We selected the three most abundant species in the data, which are also categorized as being threatened on the Norwegian Red List (Norwegian Biodiversity Information Centre 2021); *Arnica montana L*. (VU), *Fraxinus excelsior L*. (VU), and *Ulmus glabra Huds*. (EN). We further filtered any observations with a coordinate uncertainty greater than 100 m. Since these data contain no absence records and are prone to high sampling biases, we assume these data are the outcome of a thinned Poisson process from an underlying log‐Gaussian Cox Process (LGCP). We combined the species occurrence data with two environmental variables (annual mean temperature and fraction of grassland) in the ISDM to produce a map with estimated relative abundance across Norway for each species.

In addition, we supplemented the model by including two additional presence‐absence datasets (modeled as Bernoulli random variables), also obtained from GBIF, which were collected and provided by *NTNU University Museum* (Norwegian University of Science and Technology 2023) and *University of Oslo* (University of Oslo 2023). These data used standardized cross‐lists containing most vascular plants in Norway which, in addition to providing information about presences, also allowed for inference of species absences. We generated absences for these datasets by combining all the sampling locations for the species in the dataset and designating a site as absent for a species if that species was not recorded as present at that specific location.

### 2.4 Implementation of Case Study

In this section, we provide a brief example of how the *intSDM* package is used to implement our case study. We first begin the workflow by using the startWorkflow function. This requires us to specify the coordinate reference system considered, as well as the scientific names of the three species. After that, we use the $addArea function to specify the study domain of the analysis.


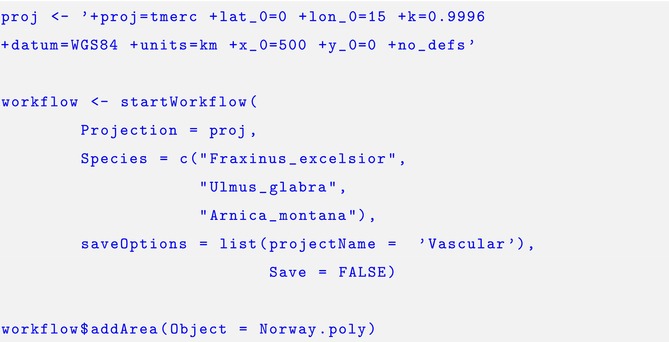


The species occurrence data may be added using the $addGBIF function. The ellipse argument (…) links to rgbif's occ_data, which allows us to filter and process the data. In this example, we specify arguments for *limit*, *coordinateUncertaintyInMeters*, and *datasetKey*, which may be found within the *GBIF documentation*.


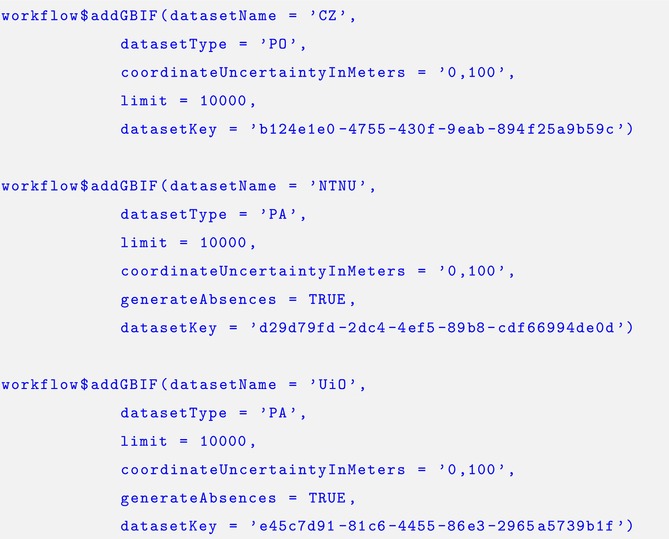


WorldClim and ESA land cover covariates are added using the. $addCovariates function. For this case study we selected the average temperature across Norway at a 2.5 min of a degree resolution, and standardized the raster data.


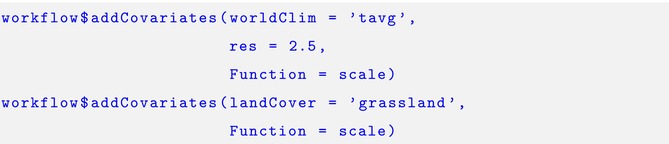


Our ISDM included a shared Gaussian random field between the datasets to account for potential spatial autocorrelation. In order to estimate this, we are required to create a mesh object, which can be done using the. $addMesh function. We used PC priors for the range and sigma parameters for the Matérn covariance function of the random field (Simpson et al. 2017), which are designed to reduce over‐fitting in the model by penalizing the model towards the base model. By doing this, we assumed that the probability of the range parameter of the covariance function being less than 200 km was 20%, and the probability that the sigma parameter was greater than 1 was 1%. This was specified using the. $specifySpatial function. To simplify the model, we set the parameter used to connect the shared spatial effect between the three likelihoods to a fixed value of unity, using the. $specifyPriors function. We also included a second random effect for the citizen science data using the. $biasFields function to reflect spatial biases in the sampling protocol, as suggested in Simmonds et al. (2020). The same PC priors were used for this spatial effect as we used for the shared spatial effect.


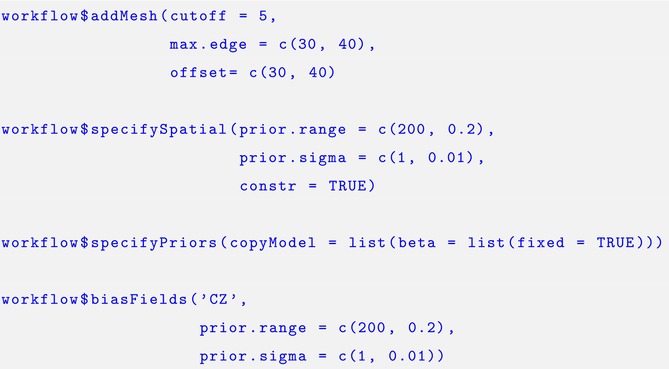


The output from this script were distribution maps reflecting the relative abundance for the three studied species and the bias fields, which was specified using the. $workflowOutput function.


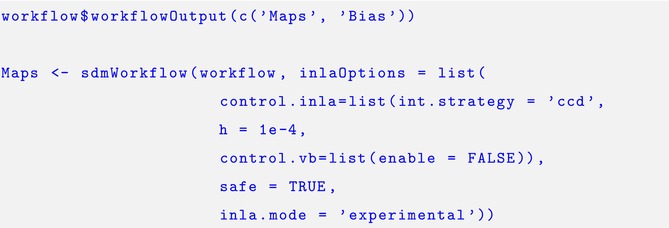


**FIGURE 3:**
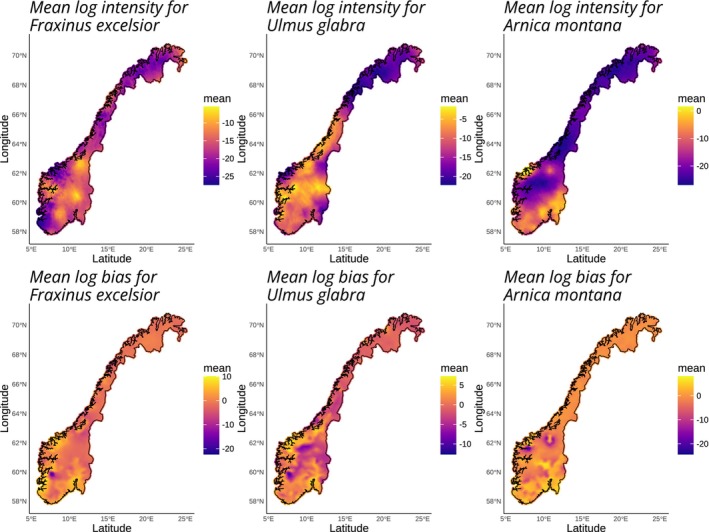
Mean of the log predicted intensity and bias for the three species, *A. montana* , *F. excelsior* , and *U. glabra* , across Norway, from ISDMs based on presence‐absence and presence‐only data for each respective species.

Finally the workflow was implemented using the sdmWorkflow function by supplying the workflow object and additional *R‐INLA* options. This function produced the maps of log‐intensity and bias illustrated by Figure 3. The predictions suggest similar distribution patterns for *Fraxinus excelsior* and *Ulmus glabra* , which have highest intensity towards the central parts of the country. On the other hand, *Arnica montana* only has high intensity along the south‐eastern coast, and very low intensity towards the north. The predicted bias fields for the three fields all look very similar, with a higher mean towards the southern part of Norway, and a lower mean towards the northern section where there were few presence‐only records collected.

## 3 Discussion

Reproducible workflows are imperative in ecology to ensure full transparency, generality, and consistency in the results of research studies. Even though it is impossible to perfectly replicate observational studies in the field, ecologists need to focus on producing reproducibility in areas of their studies where they can, such as computational reproducibility (Powers and Hampton 2019). Employing methods such as data integration for SDMs may assist in producing such workflows since they minimize the challenges related to combining observations from heterogeneous study systems.

A package like *intSDM* provides the toolkit to produce an open, reproducible, and flexible workflow integrating different data types from multiple sources to use integrated SDMs to construct maps of species distributions. Specifically, it facilitates the combination of presence‐only, presence‐absence, and counts data, which have the potential to increase the quality of SDMs. A benefit of presenting the workflow through a *CRAN*‐submitted *R* package is that certain forms of software reproducibility, such as continuous integration, adequate documentation, and unit tests, are required to ensure the package's functions and scripts run properly on a variety of platforms.

In the presented case study, we used the package to produce maps of predicted species distributions for three red‐listed vascular plant species in Norway. Such a map could provide a knowledge basis for biodiversity conservation. Furthermore, reproducible workflows like *intSDM* have the potential to implement and thus promote best practice standards in the model building process of SDMs such as those proposed by Araújo et al. (2019) and Zurell et al. (2020)), which have been sketched out in order to improve the overall transparency of reproducible workflows, raise awareness of model quality, and reduce potential misuse of model outputs.

However, future research needs to be considered for *intSDM* in order to make it more flexible and generalizable for different analyses and easier to use in the future. Incorporating different elements such as adding a temporal component to the model is required in order to understand the dynamics of species over time. In addition, creating a graphical user interface (GUI) would make the package more attractive for users with minimal coding skills, and would be able to output visual summaries of each component of our workflow. More extensive model structures should be accompanied by expanded documentation, perhaps through the form of a full step‐by‐step user manual.

The extension to this package should improve the upstream components of this workflow: obtaining data from many different online data repositories (not only GBIF), considering more data types other than abundance, presence‐only, and presence‐absence data, and being able to create a coherent data structure for each dataset with standardized metadata—even if sampling protocols vary greatly between them.

Despite the substantial added value of integrating several sources of data in SDMs, the use of disparate data may still cause some challenges. Presence‐only data are often the most abundant data source, but observations are likely to be biased towards conveniently located, densely populated areas (Sicacha‐Parada et al. 2021) or certain species (Amano et al. 2016; Troudet et al. 2017). Integrating presence‐absence and presence‐only data can increase the quality of SDMs, but how much depends on the amount of available presence‐absence data and the ability to correct for bias in the presence‐only data (Simmonds et al. 2020). Datasets of different sizes and quality affect results by producing biased estimates towards the more abundant data sources (Zipkin et al. 2021). Common solutions involve sub‐sampling, thinning, or down‐weighting the more abundant datasets; however, these methods are often subjective and can lead to different conclusions (Maunder and Piner 2017). Furthermore, while our framework accounts for the preferential sampling inherent in presence‐only data, there are numerous other biases which need to be accounted for; for example: misclassification (Wright et al. 2020) and imperfect detection (Kéry and Schmidt 2008; Richter et al. 2021).

For species with fewer occurrences, issues connected to low data availability may arise, such as inflating the uncertainty of model predictions obtained for these species. Despite this, the modeling framework here could be used as a potential solution. Plotting species intensities against rare species occurrences could be used to show where rare species are likely to be found, but have not been sampled yet, providing a good basis for more investigation and fieldwork to expand knowledge of the species distribution.

For an open workflow to be truly reproducible, data need to be open too; therefore, there is an ongoing effort towards making biodiversity data available. Data from GBIF are FAIR (Wilkinson et al. 2016) and formatted according to prevailing standards such as Darwin Core (Wieczorek et al. 2012). Despite this, there was deficient documentation and metadata for species occurrence data in our case study, requiring consultation with data owners to understand the collection methods. A lack of necessary documentation is not unique to this specific data set, highlighting that there are still challenges to resolve to make biodiversity science truly open and reproducible. Understanding the output of the model properly is essential when using a tool made by others. This is not unique for *intSDM* but rather applies to all available *R*‐packages or tools for data analyses. Providing properly documented scripts and functions and vignettes illustrating the use of *intSDM* is the first step to mitigate this issue.

It is also important to understand the data included in SDMs in order to account for weaknesses and data quality differences between the datasets, and to make sure data are properly prepared before use. Knowledge about the species or system under study is also key to identifying and including the necessary data to produce high‐quality SDMs, and to make sure that the produced output is sensible. The package presented here maximizes the usefulness of available open‐source data through integration, and provides a structured framework for other users to follow in order to continue producing open, reproducible workflows.

Finally, an area which should be looked into is the issue of communicating results to conservation practitioners and stakeholders. Metrics such as log‐intensity and log‐bias are not as understandable as more traditional habitat suitability metrics obtained by other *R* packages, notably: *dismo* (Hijmans et al. 2023), *ecospat* (Broennimann et al. 2024) and *biomod2* (Thuiller et al. 2024). Efforts should then be made to translate these quantities into something for the specific needs of practitioners, thereby ensuring that scientific insights are effectively applied in conservation strategies.

## Author Contributions

**Philip S. Mostert:** conceptualization (equal), formal analysis (lead), software (lead), visualization (equal), writing – original draft (equal), writing – review and editing (equal). **Ragnhild Bjørkås:** conceptualization (equal), writing – original draft (equal), writing – review and editing (equal). **Angeline J. H. M. Bruls:** conceptualization (equal), writing – original draft (equal), writing – review and editing (equal). **Wouter Koch:** conceptualization (equal), visualization (equal), writing – original draft (equal), writing – review and editing (equal). **Ellen C. Martin:** conceptualization (equal), writing – original draft (equal), writing – review and editing (equal). **Sam W. Perrin:** software (supporting), writing – review and editing (equal).

## Conflicts of Interest

The authors declare no conflicts of interest.

## Data Availability

All code and data used in this manuscript are freely available in the *R* package, *intSDM*, which is available on the Comprehensive R Archive Network: https://CRAN.R‐project.org/package=intSDM (Mostert et al. 2022). The *R* code is also available on GitHub: https://github.com/PhilipMostert/intSDM. The version of the package used for this manuscript (v2.1.1) is archived on Zenodo: https://doi.org/10.5281/zenodo.8430035 (Mostert et al. 2024).
